# Contribution of Phase Resetting to Statistical Persistence in Stride Intervals: A Modeling Study

**DOI:** 10.3389/fncir.2022.836121

**Published:** 2022-06-22

**Authors:** Kota Okamoto, Ippei Obayashi, Hiroshi Kokubu, Kei Senda, Kazuo Tsuchiya, Shinya Aoi

**Affiliations:** ^1^Department of Aeronautics and Astronautics, Graduate School of Engineering, Kyoto University, Kyoto Daigaku-Katsura, Kyoto, Japan; ^2^Cyber-Physical Engineering Information Research Core (Cypher), Okayama University, Okayama, Japan; ^3^Department of Mathematics, Graduate School of Science, Kyoto University, Kyoto, Japan; ^4^Department of Mechanical Science and Bioengineering, Graduate School of Engineering Science, Osaka University, Osaka, Japan

**Keywords:** human walking, stride interval fluctuation, neuromechanical model, central pattern generator, phase resetting, statistical persistence

## Abstract

Stride intervals in human walking fluctuate from one stride to the next, exhibiting statistical persistence. This statistical property is changed by aging, neural disorders, and experimental interventions. It has been hypothesized that the central nervous system is responsible for the statistical persistence. Human walking is a complex phenomenon generated through the dynamic interactions between the central nervous system and the biomechanical system. It has also been hypothesized that the statistical persistence emerges through the dynamic interactions during walking. In particular, a previous study integrated a biomechanical model composed of seven rigid links with a central pattern generator (CPG) model, which incorporated a phase resetting mechanism as sensory feedback as well as feedforward, trajectory tracking, and intermittent feedback controllers, and suggested that phase resetting contributes to the statistical persistence in stride intervals. However, the essential mechanisms remain largely unclear due to the complexity of the neuromechanical model. In this study, we reproduced the statistical persistence in stride intervals using a simplified neuromechanical model composed of a simple compass-type biomechanical model and a simple CPG model that incorporates only phase resetting and a feedforward controller. A lack of phase resetting induced a loss of statistical persistence, as observed for aging, neural disorders, and experimental interventions. These mechanisms were clarified based on the phase response characteristics of our model. These findings provide useful insight into the mechanisms responsible for the statistical persistence of stride intervals in human walking.

## 1. Introduction

Human walking is not perfectly periodic. The stride interval fluctuates from one stride to the next, exhibiting statistical persistence (Hausdorff et al., 1995; West and Griffin, 1998, 1999; Dingwell and Cusumano, 2010), which indicates that deviations in a time series are statistically more likely to be followed by subsequent deviations in the same direction. Although the stride interval fluctuations change depending on the gait speed and during development from childhood to adulthood, the statistical persistence remains unchanged (Hausdorff et al., 1996, 1999). However, the stride interval fluctuations for elderly subjects (Hausdorff et al., 1997) and patients with Huntington's disease (Hausdorff et al., 1997) or Parkinson's disease (Frenkel-Toledo et al., 2005) become uncorrelated. Experimental interventions for walking, such as the use of a metronome, also make the stride interval fluctuations uncorrelated (Hausdorff et al., 1996). It is largely unclear why statistical persistence appears in stride intervals in human walking and why this statistical property is changed by aging, neural disorders, and experimental interventions.

It has been hypothesized that the central nervous system has an underlying persistence and is responsible for the statistical persistence in stride intervals. This is supported by the finding that statistical persistence remains in patients with significant peripheral nerve degeneration (Gates and Dingwell, 2007). Various neural system models have been developed to reproduce the statistical persistence and investigate the associated mechanisms. Hausdorff et al. (1995) developed a model of the central pattern generators (CPGs) in the spinal cord and introduced “memory” into the CPG model by allowing transitions from frequency to frequency. Ashkenazy et al. (2002) extended this model by introducing a random walk for the signal transmission of neural circuits. West and Scafetta (2003) developed a “Super CPG” model that introduces external interventions *via* a forced van der Pol oscillator.

Human walking is a complex phenomenon generated through dynamic interactions between the central nervous system and the biomechanical system. It has also been hypothesized that the statistical persistence in stride intervals emerges through complex interactions during walking. Fu et al. (2020) integrated a biomechanical model composed of seven rigid links with a CPG model, which incorporated a phase resetting mechanism as sensory feedback as well as feedforward, trajectory tracking, and intermittent feedback controllers, to reproduce statistical persistence. They showed that a lack of phase resetting induces a loss of statistical persistence. However, it is difficult to fully understand the essential mechanisms responsible for generating and changing this statistical property because of the complexity of the neural and biomechanical models.

In human walking, the stance leg, which is almost straight, rotates around the foot contact point like an inverted pendulum. To investigate the essential mechanisms responsible for generating human walking from a dynamic viewpoint, simple compass-type mechanical models have been used (Kuo, 2001; Donelan et al., 2002; Kuo et al., 2005; Bruijn et al., 2011; Okamoto et al., 2020). Gates et al. (2007) and Ahn and Hogan (2013) reproduced the statistical persistence in stride intervals using simple compass-type models with sensory feedback controllers. However, they did not investigate the contribution of the feedback controllers to changes in the statistical persistence; thus, the essential mechanisms remain unclear.

The aim of this study is to clarify the contribution of phase resetting to the generation and change in the statistical persistence using a simple model. Specifically, we used a simplified neuromechanical model composed of a simple compass-type biomechanical model and a simple CPG model that incorporates phase resetting and a feedforward controller. Our model reproduced the statistical persistence in stride intervals. A lack of phase resetting induced a loss of statistical persistence, as observed in Fu et al. (2020). Furthermore, we clarified the mechanisms responsible for changes in this statistical property caused by phase resetting based on the phase response characteristics. Our findings provide important insights into the mechanisms underlying the generation and change of the statistical persistence in the stride intervals in human walking.

## 2. Methods

### 2.1. Mechanical Model

**Figure 1:**
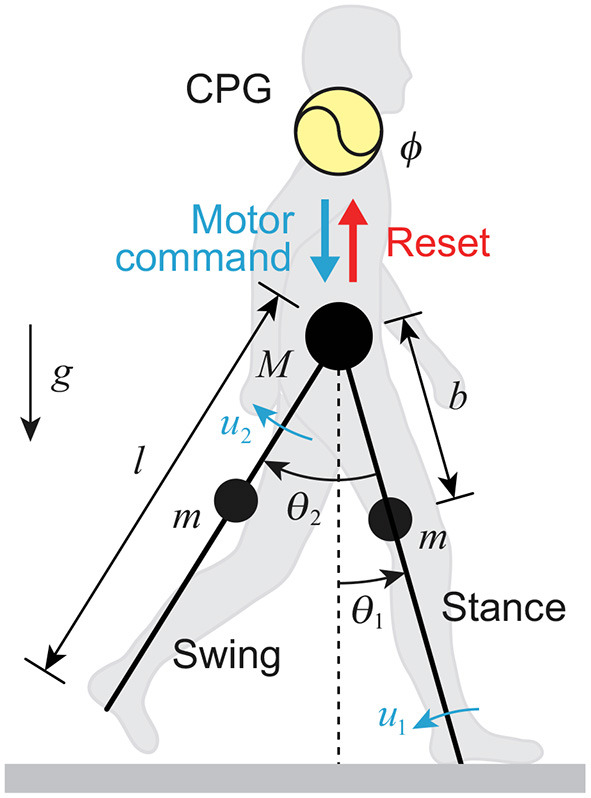
Neuromechanical model of human walking composed of CPG model with phase resetting and compass-type biomechanical model.

We used a simple compass-type model (Figure 1). This model has two legs (swing and stance legs), the lengths of which are both *l*, connected by a frictionless hip joint. The masses are located at the hip and on the legs at a distance *b* from the hip joint; *M* is the hip mass and *m* is the leg mass. θ_1_ is the angle of the stance leg with respect to the vertical, and θ_2_ is the relative angle between the stance and swing legs. The tip of the stance leg, which corresponds to the ankle, is fixed on the ground. The stance leg rotates freely without friction. This model walks on level ground *via* joint torques *u*_1_ (at the ankle) and *u*_2_ (at the hip). *g* is the acceleration due to gravity. We used the following model parameters based on Winter (2004): *M* = 50 kg, *m* = 11 kg, *l* = 1 m, *b* = 0.4 m, and *g* = 9.8 m/s^2^.

When the tip of the swing leg is in the air, the equations of motion for our model are


(1) $$\begin{array}{r} \left[\begin{array}{cc} Ml^{2}+m\left\{l^{2}+{\left(l-b\right)}^{2}\right\}-2mlb\cos\theta_{2}+mb^{2} & mlb\cos\theta_{2}-mb^{2}\\ mlb\cos\theta_{2}-mb^{2} & mb^{2} \end{array}\right]\left[\begin{array}{c} {\ddot{\theta }}_{1}\\ {\ddot{\theta }}_{2} \end{array}\right]\\ +\left[\begin{array}{c} -mlb\left({\dot{\theta }}_{2}-2{\dot{\theta }}_{1}\right){\dot{\theta }}_{2}\sin\theta_{2}\\ -mlb{\dot{\theta }}_{1}^{2}\sin\theta_{2} \end{array}\right]+\left[\begin{array}{c} -\left\{gm\left(2l-b\right)+gMl\right\}\sin\theta_{1}-gmb\sin\left(\theta_{2}-\theta_{1}\right)\\ gmb\sin\left(\theta_{2}-\theta_{1}\right) \end{array}\right]=\left[\begin{array}{c} u_{1}\\ u_{2} \end{array}\right] \end{array}$$

The tip of the swing leg touches the ground (touchdown) when the following conditions are satisfied:

(2) $$\begin{array}{r} 2\theta_{1}-\theta_{2}=0 \end{array}$$

(3) $$\begin{array}{r} \theta_{1}<0 \end{array}$$

(4) $$\begin{array}{r} 2{\dot{\theta }}_{1}-{\dot{\theta }}_{2}<0 \end{array}$$

We used condition (3) so that touchdown occurs only in front of the model to move forward, and condition (4) to ignore the scuffing of the leg tip on the ground when the swing leg is swung forward. We assumed that touchdown is a fully inelastic collision (no slip, no bounce) and that the stance leg lifts off the ground just after touchdown. Because the roles of the swing and stance legs are reversed just after touchdown, we obtain

(5) $$\begin{array}{r} \theta_{1}^{+}=-\theta_{1}^{-} \end{array}$$

(6) $$\begin{array}{r} \theta_{2}^{+}=-\theta_{2}^{-} \end{array}$$

where ∗^−^ and ∗^+^ are the state ∗ just before and after touchdown, respectively. Due to this collision, the angular velocities discontinuously change. We assumed that when the stance leg leaves the ground, it does not interact with the ground and the work of the joint torques can be neglected. These assumptions yield

(7) $$\begin{array}{r} \left[\begin{array}{c} {\dot{\theta }}_{1}^{+}\\ {\dot{\theta }}_{2}^{+} \end{array}\right]={\left\{Q^{+}\left(\theta_{1}^{-}\right)\right\}}^{-1}Q^{-}\left(\theta_{1}^{-}\right)\left[\begin{array}{c} {\dot{\theta }}_{1}^{-}\\ {\dot{\theta }}_{2}^{-} \end{array}\right] \end{array}$$

where

$$\begin{array}{l} Q^{+}\left(\theta_{1}^{-}\right)=\\ \;[\begin{array}{cc} -Ml^{2}-2m{\left(l-b\right)}^{2}-2mlb\left(1-\cos2\theta_{1}^{-}\right) & mb\left(b-l\cos2\theta_{1}^{-}\right)\\ -ml\left(b-l\cos2\theta_{1}^{-}\right) & mlb \end{array}]\\ Q^{-}\left(\theta_{1}^{-}\right)=\left[\begin{array}{cc} 2m\left(l-b\right)\left(b-l\cos2\theta_{1}^{-}\right)-Ml^{2}\cos2\theta_{1}^{-} & -m\left(l-b\right)b\\ ml\left(l-b\right) & 0 \end{array}\right] \end{array}$$

### 2.2. CPG Model

The CPGs in the spinal cord are largely responsible for rhythmic leg movements, such as during locomotion (Grillner, 1975; Shik and Orlovsky, 1976; Orlovsky et al., 1999). They can produce oscillatory behavior even in the absence of rhythmic input and sensory feedback. However, sensory feedback is crucial for producing adaptive locomotor behavior. To investigate the contribution of CPGs to adaptive locomotion in humans, various oscillator models, such as the van der Pol oscillator (Dutra et al., 2003; West and Scafetta, 2003), Matsuoka oscillator (Matsuoka, 1987; Taga et al., 1991; Taga, 1995a,b; Ogihara and Yamazaki, 2001; Hase et al., 2003; Kim et al., 2011), and phase oscillator (Yamasaki et al., 2003; Aoi et al., 2010, 2019; Dzeladini et al., 2014; Aoi and Funato, 2016; Fu et al., 2020; Tamura et al., 2020; Owaki et al., 2021), have been developed.

In this study, we used a phase oscillator, whose phase is ϕ (0 ≤ ϕ < 2π), to generate the motor commands for our model. The oscillator phase follows the dynamics expressed by

(8) $$\begin{array}{r} \dot{\phi }=\omega \end{array}$$

where ω is the basic frequency. We determined the joint torques *u*_1_ and *u*_2_ as

(9) $$\begin{array}{r} u_{1}=A_{1}\cos\phi +\sigma_{1} \end{array}$$

(10) $$\begin{array}{r} u_{2}=A_{2}\cos\left(\phi +\Delta \right)+\sigma_{2} \end{array}$$

where *A*_1_ and *A*_2_ are the amplitudes, σ_1_ and σ_2_ are noise terms, and Δ is the phase difference between *u*_1_ and *u*_2_.

It has been reported that locomotion rhythm and phase are regulated by the production of a phase shift and rhythm resetting (phase resetting) for periodic motor commands in response to sensory feedback (Lafreniere-Roula and McCrea, 2005; Rybak et al., 2006). Cutaneous feedback has been observed to contribute to phase shift and rhythm resetting behavior (Duysens, 1977; Schomburg et al., 1998). Phase resetting has thus been modeled so that the oscillator phase is reset based on foot contact information (Yamasaki et al., 2003; Aoi et al., 2010; Aoi and Funato, 2016; Fu et al., 2020; Tamura et al., 2020). In this study, we used the following relationship at touchdown:

(11) $$\begin{array}{r} \phi^{+}=\phi_{0} \end{array}$$

where ϕ_0_ is a constant. When phase resetting is not applied, ϕ is not regulated at touchdown. However, because the roles of the swing and stance legs are reversed just after touchdown so that $\theta_{i}^{+}=-\theta_{i}^{-}$ (*i* = 1, 2), we used the following relationship at touchdown:

(12) $$\begin{array}{r} \phi^{+}=\phi^{-}-\pi \end{array}$$

so that $u_{i}^{+}=-u_{i}^{-}$ (*i* = 1, 2) when the noise terms σ_1_ and σ_2_ are neglected. We designated ϕ_0_ as the value to which ϕ^+^ converged during steady walking (limit cycle) for the model without phase resetting and noise. Therefore, steady walking is identical between the models with and without phase resetting in the absence of noise. This allows us to clearly investigate the difference in the response to torque noise between cases with and without phase resetting.

This CPG model has four parameters, namely ω, *A*_1_, *A*_2_, and Δ. We used ω = 4.8 rad/s based on Hausdorff et al. (1996). Without noise (σ_1_ = σ_2_ = 0), we first investigated the dependence of gait speed during steady walking on *A*_1_, *A*_2_, and Δ, and then calculated the energy cost $\varepsilon =\int \left(u_{1}^{2}+u_{2}^{2}\right)dt$ for one step cycle for *A*_1_, *A*_2_, and Δ. We determined the parameter set (*A*_1_, *A*_2_, Δ) required to minimize ε for each gait speed. When phase resetting was used, we determined ϕ_0_ for each gait speed using the obtained parameter set.

### 2.3. Torque Noise

To simulate the stochastic fluctuation of the gait, we used two independent series of white Gaussian noise for torque noise terms σ_1_ and σ_2_ in (9) and (10), respectively, as follows:

(13) $$\begin{array}{r} \sigma_{i}=\xi U_{i}\;i=1,2 \end{array}$$

where ξ is the amplitude of the noise, and *U*_1_ and *U*_2_ are independent white Gaussian noise with standard deviation 1. This torque noise never induces consecutive touchdowns at extremely short intervals because of discontinuous and large changes in the state variables (5)–(7) at touchdown. We numerically solved the governing equations using the Euler-Maruyama method (Higham, 2001) with a time step of 10^−5^ s.

To be consistent with previous experiments on humans (Hausdorff et al., 1995, 1996, 1997), a stride was defined as two consecutive steps. Stride intervals were calculated based on the time difference between every other touchdown (strides did not overlap). Each simulation trial required the model to walk 1300 steps (650 strides). The first 150 strides were omitted from the analysis to remove transient behavior due to initial conditions.

### 2.4. Detrended Fluctuation Analysis

We used detrended fluctuation analysis (DFA) to determine the statistical persistence in the time series of stride intervals for each trial of the computer simulation. This method decreases the effect of noise and removes local trends, making it less affected by non-stationarities. The details of the method can be found elsewhere (e.g., Peng et al., 1993, 1994a,b; Hausdorff et al., 1995; Hardstone et al., 2012; Ihlen, 2012). Briefly, the feature amount *F*(*n*) constructed from segments of length *n* of the time series exhibits a power-law relationship, indicating the presence of scaling as *F*(*n*) ~ *n*^α^. We investigate the scaling exponent α to determine the statistical persistence for the time series data.

In this study, we first formed the following accumulated sum using the sequence of stride intervals *x*(*i*) for *i* = 1, 2, …, *N*, where *N* is the total number of strides (*N* = 500):

(14) $$\begin{array}{r} y\left(i\right)=\overset{i}{\underset{k=1}{\sum }}\left[x\left(k\right)-\bar{x}\right]\;i=1,2,\ldots ,N \end{array}$$

where $\bar{x}$ is the mean stride interval from *x*(1) to *x*(*N*). We then divided the integrated series *y*(*i*) into segments of length *n* (*n* < *N*), *y*_*j*_(*s*) (*j* = 1, 2, …, *N*/*n, s* = 1, 2, …, *n*), so that each segment is equal in length and non-overlapping. We next detrended each segment *y*_*j*_(*s*) by subtracting a least squares linear regression line ŷ_*j*_(*s*) fit to *y*_*j*_(*s*), and averaged the squares of the detrended data (i.e., the residuals). We thus obtained the standard deviation *F*(*n*) as

(15) $$\begin{array}{r} F\left(n\right)=\sqrt{\frac{1}{n}\overset{n}{\underset{s=1}{\sum }}{\left[y_{j}\left(s\right)-{ŷ}_{j}\left(s\right)\right]}^{2}} \end{array}$$

We used a set of *n* distributed equally on a logarithmic scale between 4 and *N*/4 (Jordan et al., 2006), specifically, *n* = 4, 5, 6, …, 87, 104, and 125 (sample size is 20).

In general, *F*(*n*) increases with increasing *n* and a graph of log *F*(*n*) vs. log *n* exhibits a power-law relationship, indicating the presence of scaling as *F*(*n*) ~ *n*^α^. We fit log *F*(*n*) vs. log *n* plots with a linear function using a standard least squares regression approach, and obtained the scaling exponent α from the slope of this line. In particular, α = 0.5 indicates that the stride intervals are completely uncorrelated (i.e., white noise). That is, DFA will still produce α = 0.5 even if the time series is rearranged in any manner (through surrogate data analysis). In contrast, α < 0.5 indicates statistical anti-persistence in stride intervals and 0.5 < α ≤ 1.0 indicates statistical persistence. When α > 1.0, the time series is brown noise (i.e., integrated white noise) (Hausdorff et al., 1995).

## 3. Results

### 3.1. Determination of Parameters for Each Gait Speed

**Figure 2:**
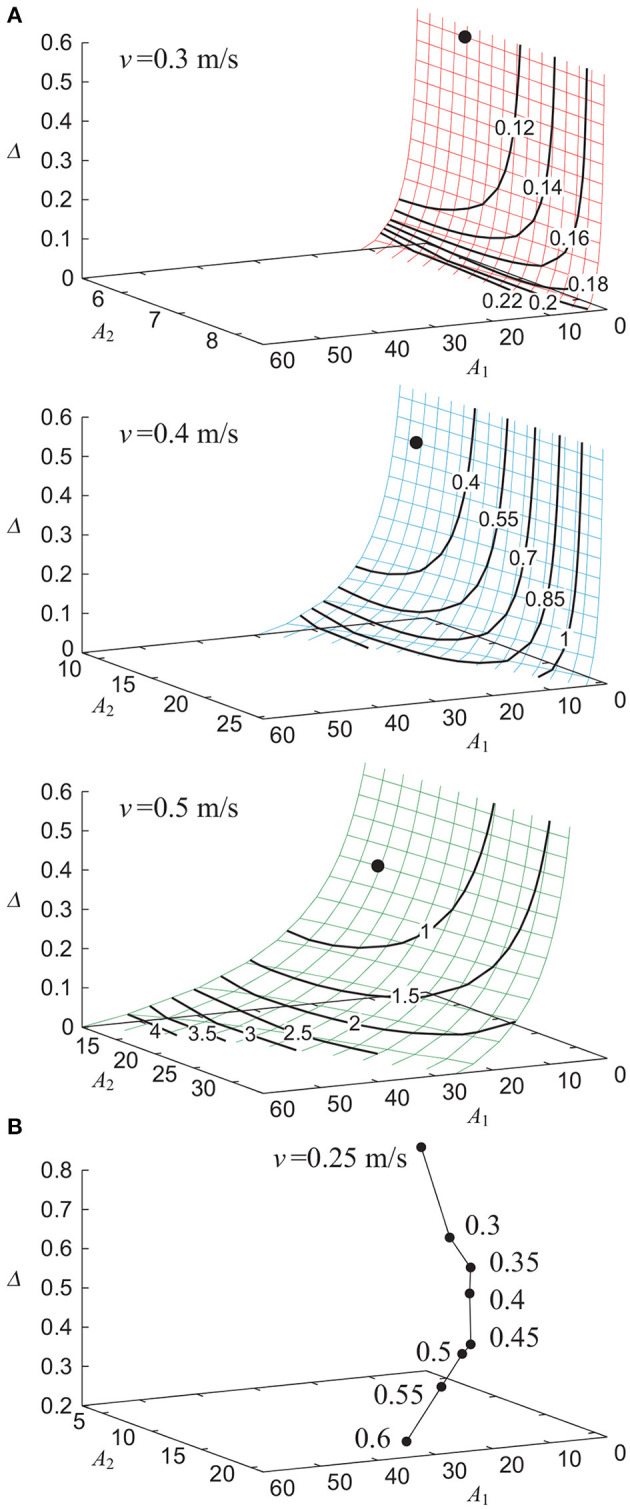
Dependence of gait performance on parameters *A*_1_, *A*_2_, and Δ without noise. **(A)** Contour of evaluation criterion ε for parameters that generate gait speed *v* = 0.3, 0.4, and 0.5 m/s. Data point indicates the parameter set that minimizes ε. **(B)** Parameter sets that minimize ε for each gait speed *v*.

Without noise (ξ = 0), our model achieved stable walking with a gait speed *v* of 0.25 to 0.6 m/s depending on the parameters *A*_1_, *A*_2_, and Δ. Figure 2A shows the contour of the evaluation criterion ε for *A*_1_, *A*_2_, and Δ, which generated *v* = 0.3, 0.4, and 0.5 m/s. Figure 2B shows the parameter sets (*A*_1_, *A*_2_, Δ), each of which minimized ε for a given gait speed *v*. The use of phase resetting did not affect these results. We use the parameter set *A*_1_ = *A*_1_(*v*), *A*_2_ = *A*_2_(*v*), and Δ = Δ(*v*) in the following sections.

### 3.2. Stride Interval Fluctuations

**Figure 3:**
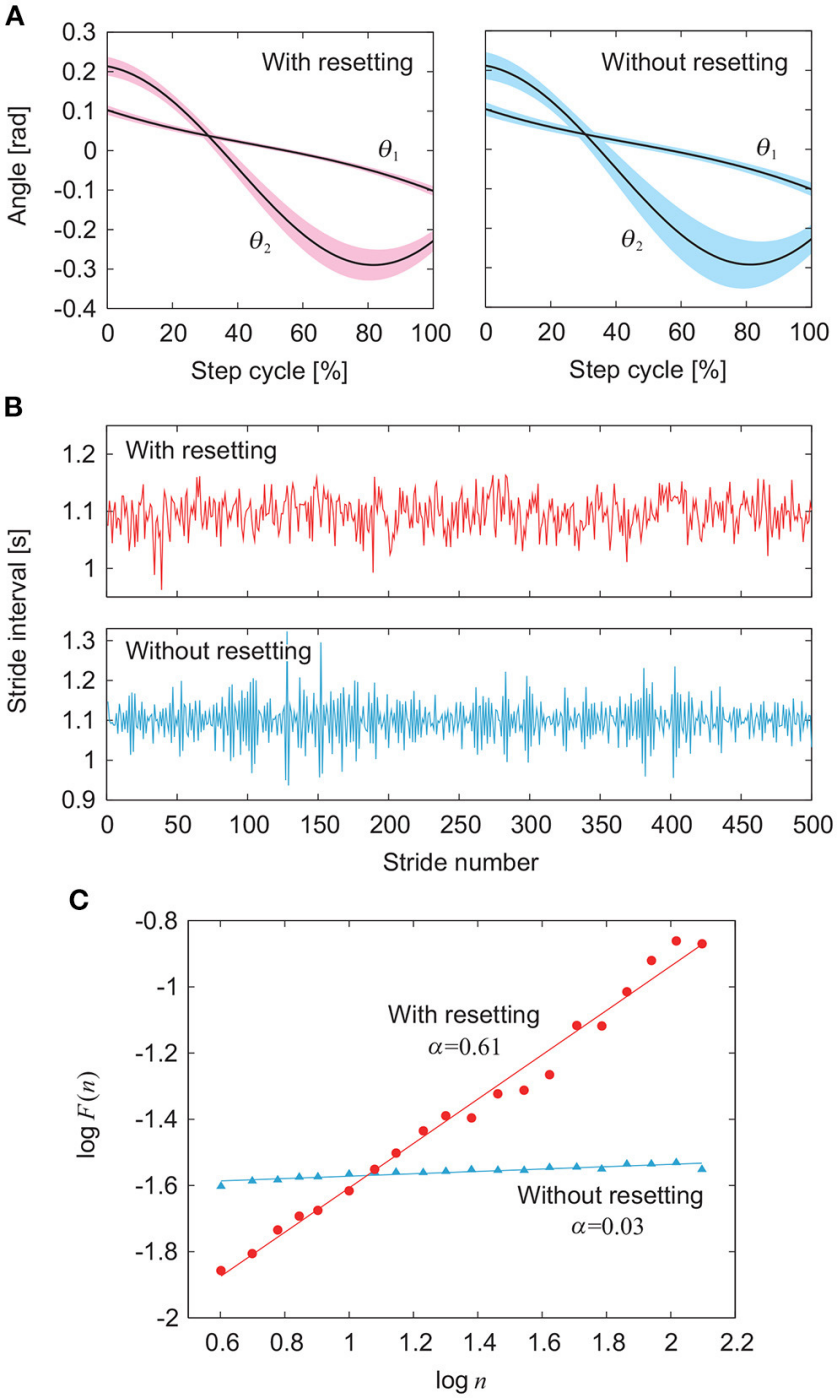
Comparison of gait fluctuations between models with and without phase resetting at gait speed *v* = 0.4 m/s using noise amplitude ξ = 1 (see Supplementary Movie). **(A)** Angles θ_1_ and θ_2_. Black lines and colored areas indicate the average and standard deviation, respectively. **(B)** Stride intervals. **(C)** Plot of log *F*(*n*) for log *n* and scaling exponent α obtained from slope of fitted line.

**Figure 4:**
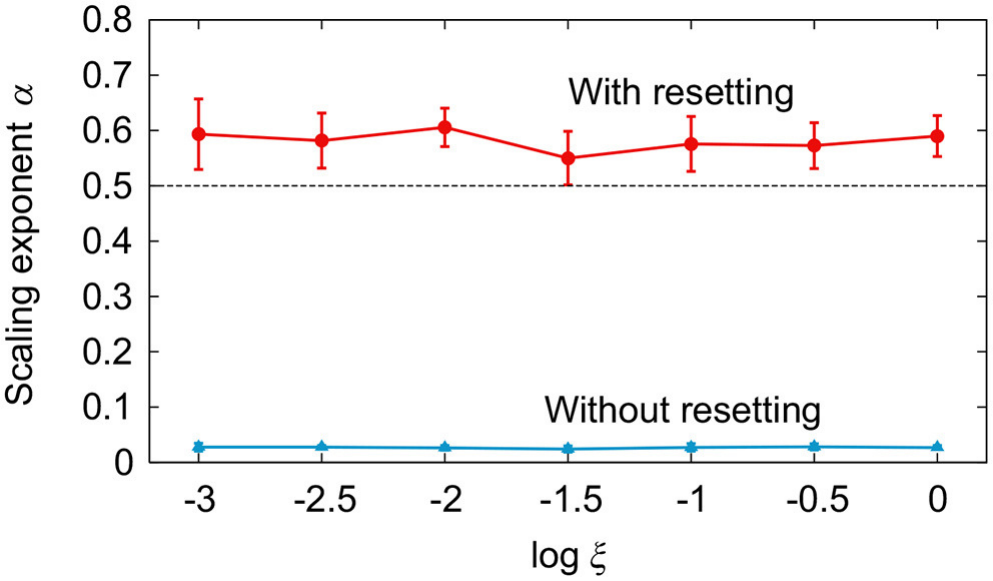
Comparison of scaling exponent α for noise amplitude ξ between models with and without phase resetting at gait speed *v* = 0.4 m/s. Data points and error bars correspond to the means and standard deviations, respectively, of the results of 10 simulations.

Figure 3 compares the simulation results between the models with and without phase resetting at a walking speed of 0.4 m/s (*A*_1_ = 4.9, *A*_2_ = 10, Δ = 0.47) using the noise amplitude ξ = 1. Figures 3A,B show the angles θ_1_ and θ_2_ and the stride intervals, respectively, during 500 strides. Although ξ is identical between the models, the model without phase resetting has larger stride interval fluctuations than those for the model with phase resetting. Figure 3C shows a plot of log *F*(*n*) for log *n* and the scaling exponent α obtained from the slope of the fitted line. The model with phase resetting exhibits statistical persistence in stride intervals (0.5 < α ≤ 1.0), which is consistent with observations of healthy adults (Hausdorff et al., 1995). Furthermore, the standard deviation of stride interval fluctuations of the model with phase resetting is 0.03, which is also consistent with observations of healthy adults (Hausdorff et al., 1995). In contrast, the model without phase resetting exhibits statistical anti-persistence in stride intervals (α < 0.5). Figure 4 shows the dependence of α on ξ. The models with and without phase resetting, both of which kept walking when ξ ≤ 1, exhibited statistical persistence and anti-persistence, respectively, regardless of ξ.

**Figure 5:**
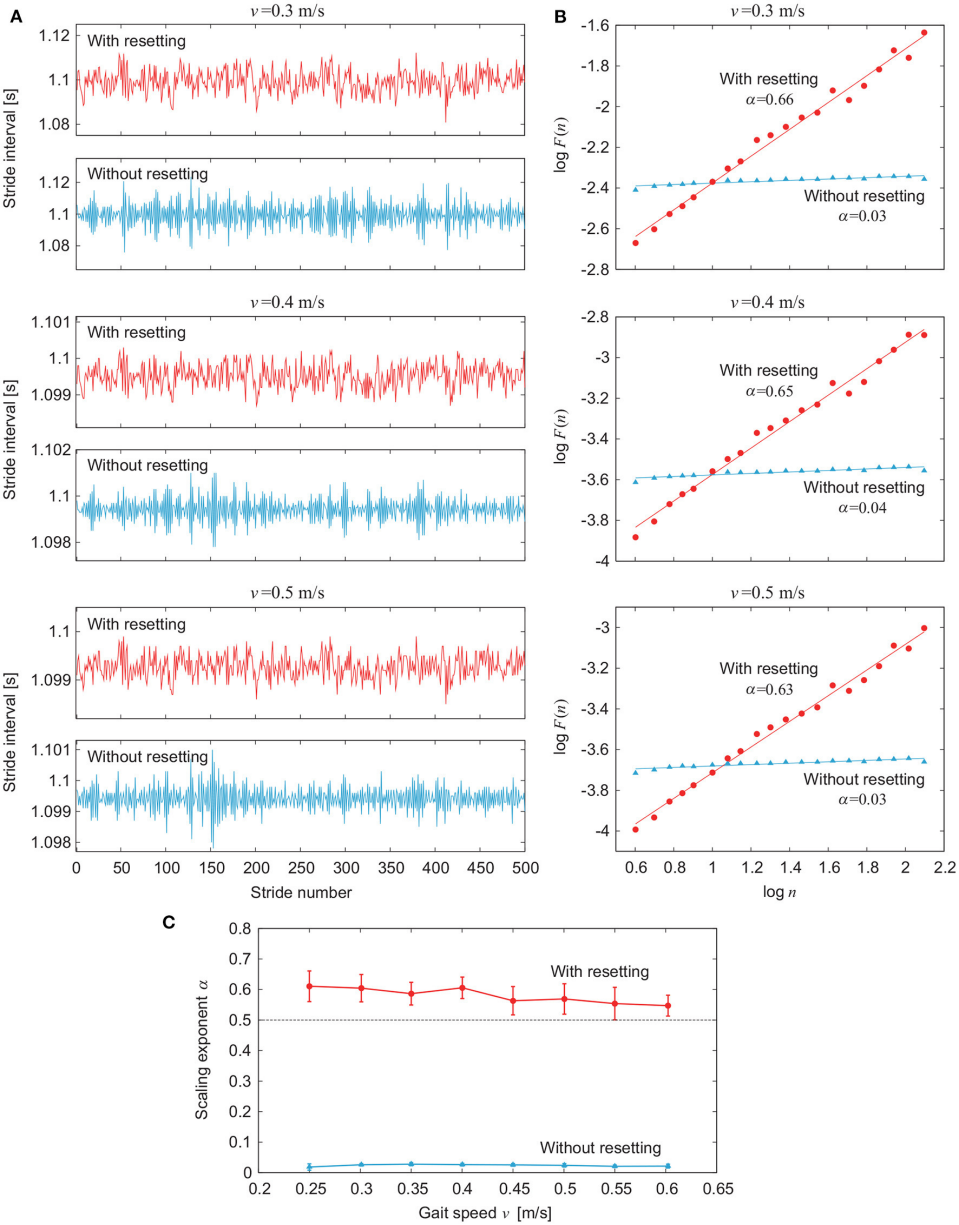
Comparison of stride interval fluctuations for various values of gait speed *v* between models with and without phase resetting using noise amplitude ξ = 10^−2^. **(A)** Stride intervals and **(B)** plot of log *F*(*n*) for log *n* for gait speed *v* = 0.3, 0.4, and 0.5 m/s. **(C)** Scaling exponent α vs. gait speed *v*. Data points and error bars correspond to the means and standard deviations, respectively, of the results of 10 simulations.

Figure 5 compares the simulation results for the models with and without phase resetting for various values of gait speed *v* using ξ = 10^−2^. Figures 5A,B show the stride intervals and log *F*(*n*) plot, respectively, for *v* = 0.3 m/s (*A*_1_ = 1.3, *A*_2_ = 6.1, Δ = 0.57), 0.4 m/s (*A*_1_ = 4.9, *A*_2_ = 10, Δ = 0.47), and 0.5 m/s (*A*_1_ = 14, *A*_2_ = 15, Δ = 0.37). Figure 5C shows the dependence of α on *v*. The model with phase resetting exhibits statistical persistence regardless of *v*, which is consistent with observations of healthy adults (Hausdorff et al., 1996). In contrast, the model without phase resetting exhibits statistical anti-persistence regardless of *v*.

## 4. Discussion

### 4.1. Mechanisms for Statistical Persistence and Anti-persistence of Stride Intervals

In this study, the model with phase resetting exhibited statistical persistence in stride intervals (0.5 < α ≤ 1.0), whereas the model without phase resetting exhibited statistical anti-persistence (α < 0.5) (Figure 3), as observed in a previous modeling study (Fu et al., 2020). Statistical anti-persistence is characterized by the alternation of large and small values. Fu et al. (2020) performed a linearized stability analysis on a model without phase resetting and noise, and showed that the dominant mode (least stable mode) characterized by Floquet multipliers was a pair of complex conjugates whose amplitude was less than but close to unity and whose argument was greater than π/2. This suggests that the fluctuation ξ_*n*_ of the stride number *n* can be approximately written as $\xi_{n}={\left(-r\right)}^{n}\xi_{1}$, where *r* ~ 1 (*r* < 1) and ξ_1_ is an initial deviation, corresponding to a slowly damped period-2 oscillation. They explained that this period-2 oscillation induced the alternation of long and short stride intervals and statistical anti-persistence. Although we performed the same stability analysis for our model, the dominant mode of our model without phase resetting and noise was positive real, whose amplitude is less than 1, indicating that the initial deviation monotonically decreases. In addition, our model with phase resetting had almost the same dominant mode as that for our model without phase resetting and it is difficult to conclude that these stability characteristics explain the difference in the statistical properties in stride intervals between the models with and without phase resetting. Furthermore, the amplitude of our dominant mode was 0.65 and the damping was relatively fast.

**Figure 6:**
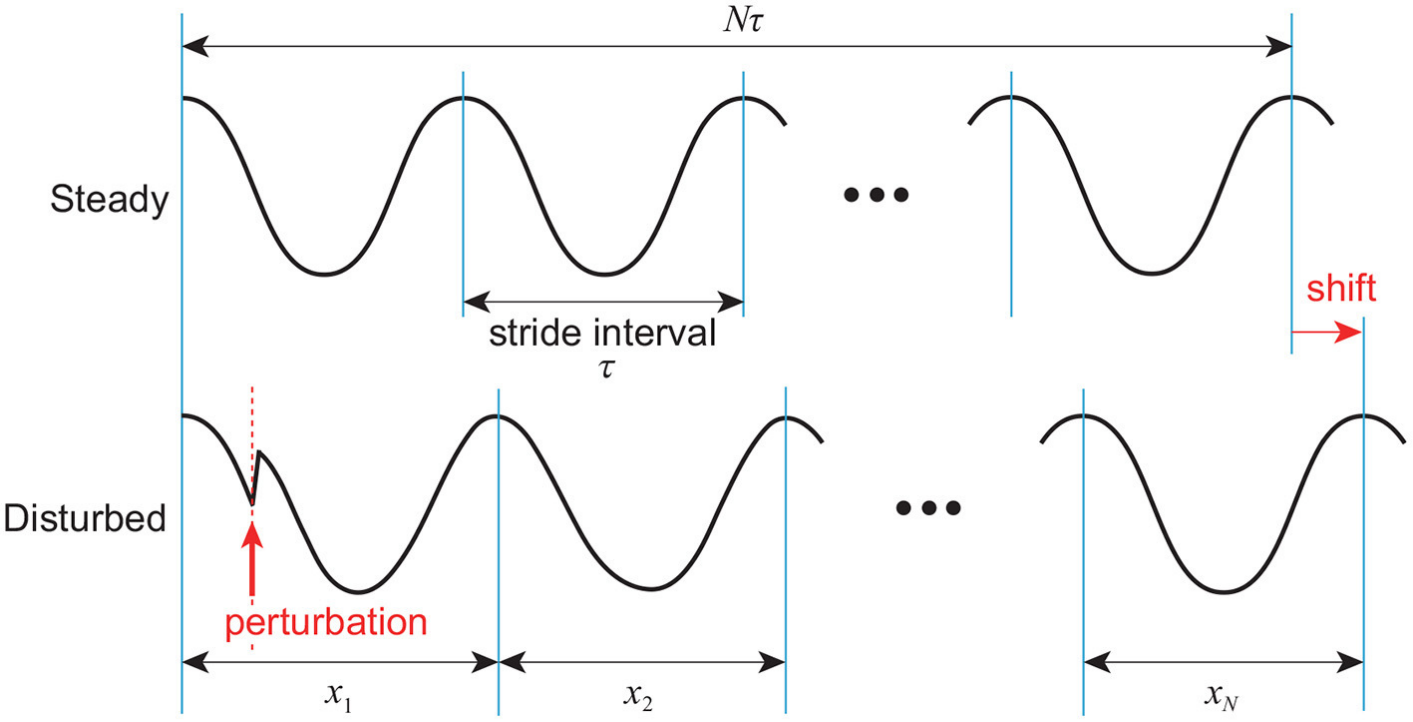
Phase shift caused by disturbance to limit cycle of walking. After recovery, locomotion phase is shifted (*x*_1_ + ⋯ + *x*_*N*_ > *Nτ*).

**Figure 7:**
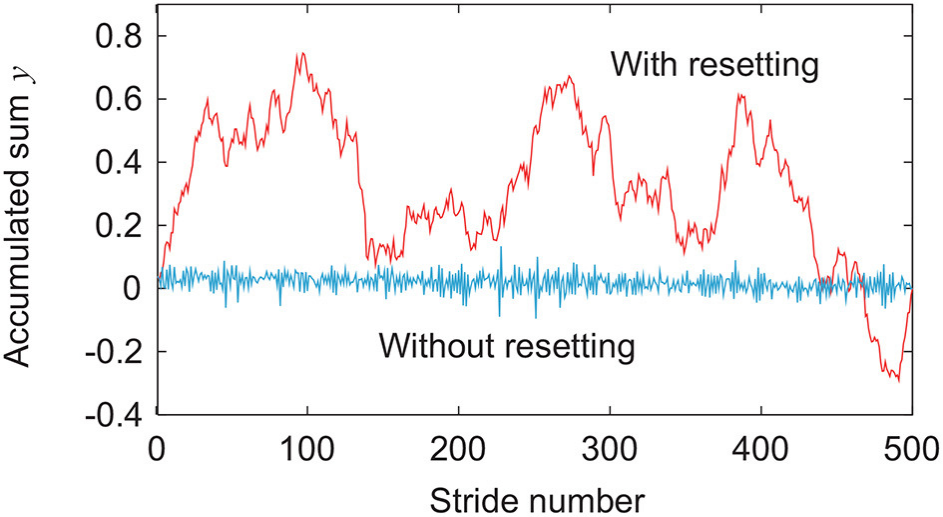
Comparison of accumulated sum *y* of stride intervals between models with and without phase resetting at gait speed *v* = 0.4 m/s and using noise amplitude ξ = 1 in Figure 3.

Next, we directly consider the difference in the response of the stride interval to disturbances. Specifically, we focus on the phase response curve in phase reduction theory (Winfree, 1980; Kuramoto, 1984), which explains how the phase of a limit cycle oscillator shifts by a perturbation at an arbitrary phase (Figure 6). The model with phase resetting shows a shift of the locomotion phase after the recovery due to phase resetting in (11) at foot contact, whereas the model without phase resetting shows no phase shift (Tamura et al., 2020). Furthermore, the phase shift for the model with phase resetting varies depending on the timing of the disturbance. Therefore, the accumulated sum *y* of stride intervals in (14) tends to move to the cumulative sum of the amount of phase shifts induced by input noise in the model with phase resetting, which results in a relatively smooth signal with large low-frequency components, as shown in Figure 7. In contrast, *y* tends to converge to 0 in the model without phase resetting, which results in a rough signal with large high-frequency components. Because the scaling exponent α increases with the degree of smoothness (Eke et al., 2000), this difference induces the difference in the scaling exponent α and statistical properties between the models with and without phase resetting.

### 4.2. Biological Relevance of Our Findings

The scaling exponent α greatly decreases during walking to a metronome in humans (Hausdorff et al., 1996), where the stride interval is constrained by an external cadence (i.e., metronome). This corresponds to the walking of the model without phase resetting, where the stride interval is constrained by the frequency ω in (8) of the phase oscillator. Therefore, the locomotion phase remains almost unchanged during walking to a metronome, and α decreases as in the model without phase resetting (Figure 3), as discussed in Section 4.1. It has been reported that α also greatly decreases for the stride interval fluctuations of elderly subjects (Hausdorff et al., 1997) and patients with Huntington's disease (Hausdorff et al., 1997) or Parkinson's disease (Frenkel-Toledo et al., 2005). Although the phase response characteristics have been clarified during walking for healthy adults (Yamasaki et al., 2003; Funato et al., 2016; Nessler et al., 2016), those during walking for elderly subjects and patients with neural disorders remain unclear. Investigating them would help clarify the mechanisms responsible for changes in the statistical persistence caused by aging and neural disorders.

Although stride interval fluctuations change depending on gait speed in humans, the statistical persistence remains unchanged (Hausdorff et al., 1996). Our model with phase resetting also exhibited statistical persistence regardless of the gait speed (Figure 5). The constraint on gait rhythm seems more crucial for the statistical persistence than the constraint on gait speed, as observed for walking to a metronome (Hausdorff et al., 1996).

The standard deviation of stride interval fluctuations is about 0.04 s in human walking, which is 3% of the mean stride interval (Hausdorff et al., 1995). It was difficult for previous studies (Gates et al., 2007; Fu et al., 2020) using biomechanical models to reproduce a magnitude of stride interval fluctuations similar to that for humans. Although Gates et al. (2007) reproduced statistical persistence in stride intervals (0.5 < α ≤ 1.0) using a simple biomechanical model as in this study, their model was not robust and the noise amplitude was limited. Therefore, their stride interval fluctuations were much smaller than those in humans. Furthermore, the scaling exponent α was sensitive to the noise amplitude, and the fluctuations exhibited brown noise at high noise levels (α > 1.0). In contrast, phase resetting made our model robust, which allowed a magnitude of stride interval fluctuations similar to that for healthy adults (Figure 3). Furthermore, α was 0.5 to 1.0, which is consistent with observations of healthy adults, and was not sensitive to the noise amplitude (Figure 4), but sensitive to the controller (i.e., whether phase resetting was used).

Previous studies (Yamasaki et al., 2003; Aoi et al., 2010; Fujiki et al., 2018; Tamura et al., 2020) have shown that phase resetting contributes to adaptive walking. In this study, we found that it also contributes to the statistical persistence of gait. In addition to the fact that statistical persistence is impaired by aging (Hausdorff et al., 1997), central nervous system diseases, such as Parkinson's disease (Frenkel-Toledo et al., 2005) and Huntington's disease (Hausdorff et al., 1997), and experimental intervention for walking (Hausdorff et al., 1996), it has been suggested that statistical persistence is linked to important characteristics of gait. Bohnsack-McLagan et al. (2016) suggested that fluctuation persistence leads to redundancies in gait and helps predict and prevent fall risk. Ahn and Hogan (2013) and Fu et al. (2020) showed that fluctuation persistence appears in gait with low gait stability. Gates et al. (2007) showed that a decrease in the ability to perform finely controlled movements leads to an increase in motor output noise and impairs the persistence of fluctuations.

Many studies have reported long-range correlations in stride intervals in human walking based on the results of DFA (Hausdorff et al., 1995, 1996, 1997; Ashkenazy et al., 2002), which indicates that stride-to-stride correlations decay in a scale-free (fractal-like) power-law fashion and suggests that each stride depends explicitly on many previous strides. However, DFA is highly sensitive to yielding false positive results (Maraun et al., 2004; Höll and Kantz, 2015), and it is difficult to conclude the presence of long-range correlations from DFA alone. Instead, DFA provides a valid indicator of statistical persistence and anti-persistence in a time series (Maraun et al., 2004). In this study, we used statistical persistence instead of long-range correlations to interpret the results of DFA, as discussed in Dingwell and Cusumano (2010).

### 4.3. Limitations of Our Model and Future Work

Based on the hypothesis that the statistical persistence in stride intervals emerge through dynamic interactions between the neural and biomechanical systems, we integrated a simple neural model and a simple biomechanical model to reproduce statistical persistence in stride intervals and change in this statistical property. However, our model is very simple and has limitations with regard to replicating many aspects of human walking. In particular, because the feedforward torques (9) and (10) were simply composed of a sinusoidal wave, the gait speeds of our model were slower than those of healthy adults (Figure 2). In addition, although statistical persistence could be associated with low gait stability (low convergence speed to the limit cycle) (Ahn and Hogan, 2013; Fu et al., 2020), our model had higher stability than that of complicated models due to its simplicity. The high stability of our model with phase resetting might have caused the scaling exponent α to be ~ 0.6, which is smaller than that (~ 1) in healthy adults (Hausdorff et al., 1995). Furthermore, stochastic noise is ubiquitous in the central nervous system and peripheral sensory-motor systems (Jones et al., 2002; van Beers et al., 2004; Churchland et al., 2006). However, our model used only torque noise, which may result in the difference between the statistical anti-persistence in the model without phase resetting and the white noise in walking to a metronome in humans (Hausdorff et al., 1996; Bohnsack-McLagan et al., 2016).

Based on the findings in this study, it is important to verify the essential mechanisms responsible for changes in the statistical persistence by using biologically detailed neuromusculoskeletal models. In a previous study (Tamura et al., 2020), we integrated a musculoskeletal model composed of seven rigid links and 18 muscles with a CPG model with a muscle synergy-based controller to investigate the contribution of phase resetting to the phase response characteristics during walking. In another previous study (Fujiki et al., 2019), we used a half-center type CPG model composed of a rhythm generator network, which was modeled using neuron populations of flexor and extensor centers based on Danner et al. (2016, 2017) and Rybak et al. (2006), to clarify the mechanisms responsible for the CPG responses to afferent stimulation using dynamic systems theory based on nullclines. We plan to incorporate these biologically detailed models to further investigate the mechanisms responsible for changes in the statistical persistence.

## 5. Conclusion

In this study, we clarified the contribution of phase resetting to the generation and change of statistical persistence using a simple neuromechanical model. Specifically, our model reproduced the statistical persistence in stride intervals. A lack of phase resetting induced a loss of statistical persistence. Furthermore, we clarified the mechanisms responsible for changes in statistical persistence caused by phase resetting based on the phase response characteristics. Our findings provide important insight into the mechanisms underlying the generation and change of the statistical persistence in the stride intervals in human walking.

## Data Availability Statement

The raw data supporting the conclusions of this article will be made available by the authors, without undue reservation.

## Author Contributions

SA developed the study design. KO performed simulation experiments and analyzed the data in consultation with SA, IO, HK, KS, and KT. KO and SA wrote the manuscript. All authors reviewed and approved it.

## Funding

This study was supported in part by JSPS KAKENHI Grant Numbers JP21J23164 and JP20H00229; and JST FOREST Program Grant Number JPMJFR2021.

## Conflict of Interest

The authors declare that the research was conducted in the absence of any commercial or financial relationships that could be construed as a potential conflict of interest. The reviewer TN declared a shared affiliation with the author SA to the handling editor at time of review.

## Publisher's Note

All claims expressed in this article are solely those of the authors and do not necessarily represent those of their affiliated organizations, or those of the publisher, the editors and the reviewers. Any product that may be evaluated in this article, or claim that may be made by its manufacturer, is not guaranteed or endorsed by the publisher.
